# Systematic assessment of HER2 status in ductal carcinoma in situ of the breast: a perspective on the potential clinical relevance

**DOI:** 10.1186/s13058-024-01875-w

**Published:** 2024-08-27

**Authors:** Mieke R. Van Bockstal, Jelle Wesseling, Ester H. Lips, Marjolein Smidt, Christine Galant, Carolien H. M. van Deurzen

**Affiliations:** 1 Department of Pathology, Cliniques universitaires Saint-Luc, Avenue Hippocrate 10, 1200 Brussels, Belgium; 2 Pôle de Morphologie (MORF), Institut de Recherche Expérimentale et Clinique, Université catholique de Louvain, Avenue Hippocrate 10, 1200 Brussels, Belgium; 3 Division of Molecular Pathology, The Netherlands Cancer Institute, Plesmanlaan 121, 1066CX, Amsterdam, The Netherlands; 4 Department of Pathology, The Netherlands Cancer Institute – Antoni van Leeuwenhoek, Plesmanlaan 121, 1066CX, Amsterdam, The Netherlands; 5 Department of Pathology, Leiden University Medical Centre, P.O. Box 9600, 2300 RC Leiden, The Netherlands; 6 Department of Surgery, Maastricht University Medical Center, P.O. Box 616, 6200 MD Maastricht, The Netherlands; 7 Department of Pathology, Erasmus MC Cancer Institute Rotterdam, Doctor Molewaterplein 40, 3015 GD Rotterdam, The Netherlands

**Keywords:** Ductal carcinoma in situ, HER2, Immunohistochemistry, Breast, Grade, Hormone receptor status, Biopsy, Nuclear atypia, Personalized therapy

## Abstract

In many countries, hormone receptor status assessment of ductal carcinoma in situ (DCIS) is routinely performed, as hormone receptor-positive DCIS patients are eligible for adjuvant anti-hormonal treatment, aiming to reduce the ipsilateral and contralateral breast cancer risk. Although *HER2* gene amplification and its associated HER2 protein overexpression constitute a major prognostic and predictive marker in invasive breast carcinoma, its use in the diagnosis and treatment of DCIS is less straightforward. HER2 immunohistochemistry is not routinely performed yet, as the role of HER2-positivity in DCIS biology is unclear. Nonetheless, recent data challenge this practice. Here, we discuss the value of routine HER2 assessment for DCIS. HER2-positivity correlates strongly with DCIS grade: around four in five HER2-positive DCIS show high grade atypia. As morphological DCIS grading is prone to interobserver variability, HER2 immunohistochemistry could render grading more robust. Several studies showed an association between HER2-positive DCIS and ipsilateral recurrence risk, albeit currently unclear whether this is for overall, in situ or invasive recurrence. HER2-positive DCIS tends to be larger, with a higher risk of involved surgical margins. HER2-positive DCIS patients benefit more from adjuvant radiotherapy: it substantially decreases the local recurrence risk after lumpectomy, without impact on overall survival. HER2-positivity in pure biopsy-diagnosed DCIS is associated with increased upstaging to invasive carcinoma after surgery. HER2 immunohistochemistry on preoperative biopsies might therefore provide useful information to surgeons, favoring wider excisions. The time seems right to consider DCIS subtype-dependent treatment, comprising appropriate local treatment for HER2-positive DCIS patients and de-escalation for hormone receptor-positive, HER2-negative DCIS patients.

## Introduction

**Fig. 1:**
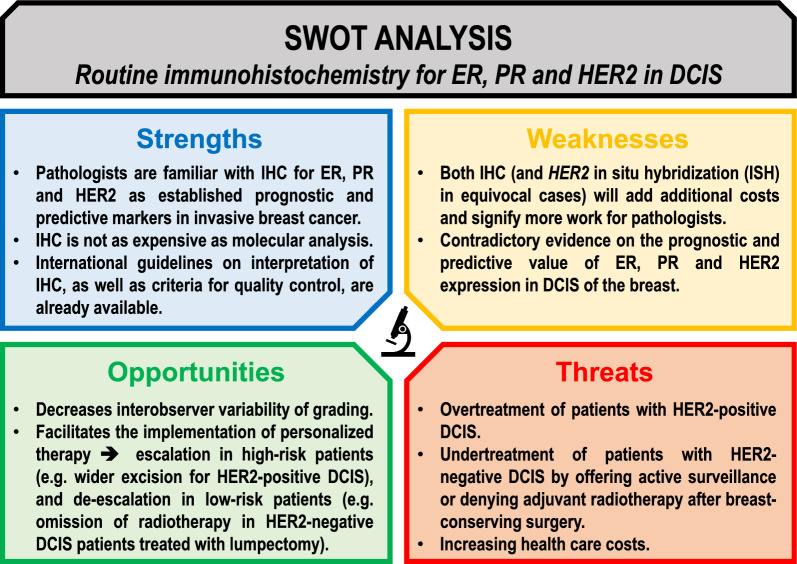
Strength, Weakness, Opportunity and Threat (SWOT) analysis for systematic immunohistochemistry in DCIS. Routine assessment of hormone receptor status and HER2 protein expression by systematic immunohistochemical analysis in DCIS could increase interobserver concordance of DCIS grading. HER2 status may be used to personalize treatment, with de-escalation of therapy in HER2-negative DCIS patients

Ductal carcinoma in situ (DCIS) is regarded as a non-obligate precursor lesion of invasive breast carcinoma (IBC), with marked heterogeneity at the morphological, immunohistochemical and molecular level [1]. Histopathological grading of DCIS is prone to substantial interobserver variability, with kappa statistics ranging from 0.27 to 0.67, irrespective of the classification system used [2]. Notwithstanding the histological grade, most DCIS patients are uniformly treated, either by lumpectomy and radiotherapy or by mastectomy, depending on the tumor size and the breast size, and ultimately, the patients’ preferences. In several countries, national guidelines recommend hormone receptor status assessment since adjuvant treatment with tamoxifen or aromatase inhibitors in hormone receptor-positive DCIS reduces both the ipsilateral recurrence risk and the contralateral breast cancer risk [3–5]. However, systematic immunohistochemistry (IHC) for estrogen receptor (ER), progesterone receptor (PR) and HER2 for so-called ‘surrogate molecular subtyping’ is currently only performed for IBC. In particular, the HER2 status in DCIS is not routinely assessed yet, because its role in tumor biology is unclear and there seems to be no substantial clinical impact so far. In the present ‘perspective’, we discuss why it could be useful to add HER2 assessment to hormone receptor status assessment in the pre-operative DCIS work-up. Figure 1 provides an overview of all potential advantages and disadvantages of systematic IHC of ER, PR and HER2 in DCIS. We address this issue through several questions, most of them still debated, which could help stimulate research efforts in these different fields, and pave the way towards a DCIS subtype-dependent treatment. This perspective article does not comprise an exhaustive systematic review nor meta-analysis, but we aimed to provide an evidence-based plea for routine implementation of HER2 IHC in DCIS.

## Why is DCIS more often HER2-positive than IBC?

**Fig. 2:**
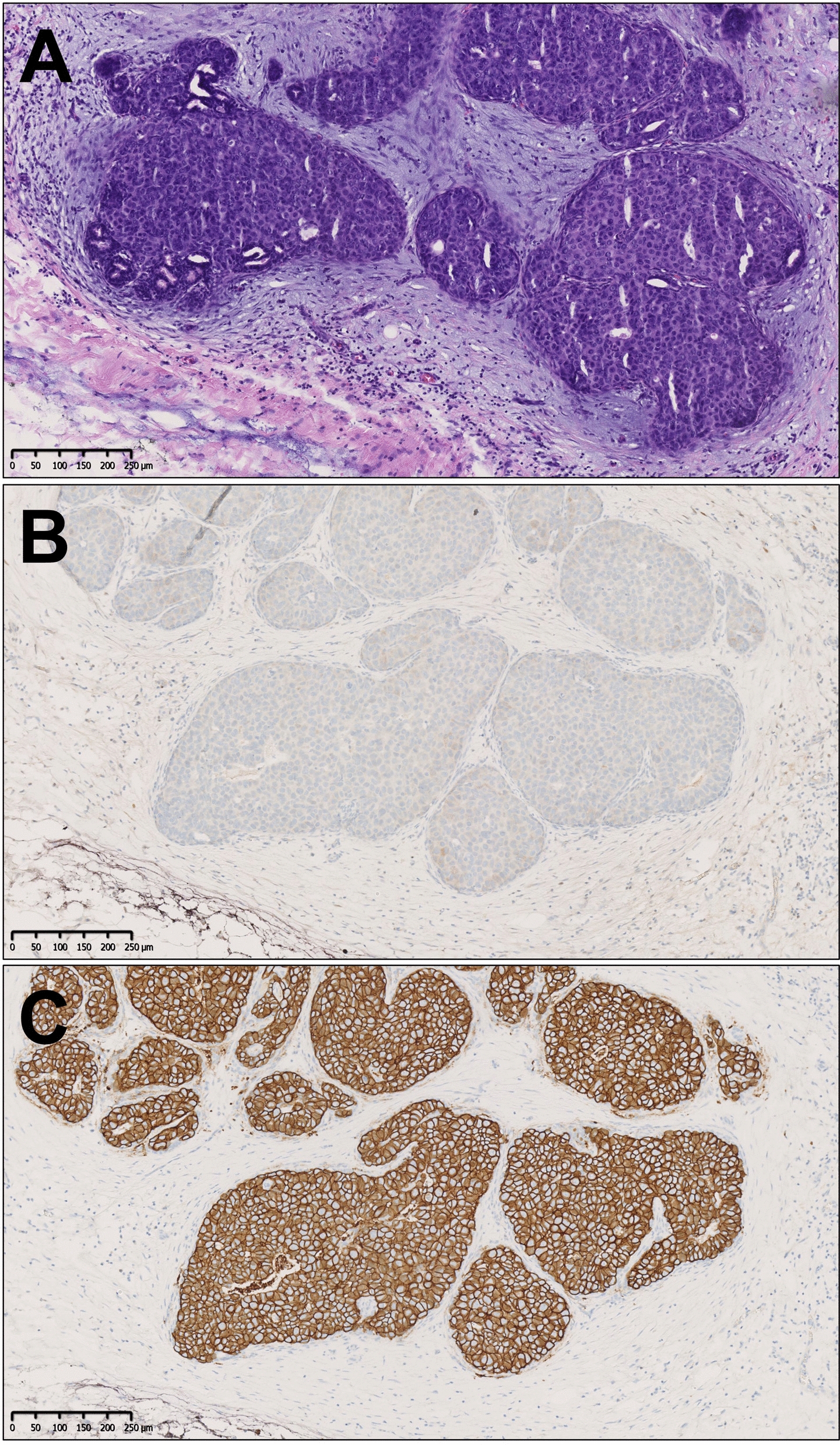
Example of a HER2-positive DCIS. Hematoxylin and eosin stain of an intermediate grade ductal carcinoma in situ (DCIS), surrounded by myxoid stroma (**A**). This DCIS shows no nuclear estrogen receptor expression (**B**) and diffuse strong circumferential membrane staining for HER2 (**C**). Original magnification 100x

*HER2* gene amplification and its associated HER2 protein overexpression occur in around 14% of IBC [6], wherein it correlates with aggressive behavior and poor prognosis in the absence of targeted therapy. As such, HER2-positivity constitutes an important predictive marker for anti-HER2 drugs [7]. Paradoxically, HER2-positivity is much more common in DCIS (Fig. 2), with a prevalence ranging from 27 to 35% in several large study cohorts [8–12]. If DCIS would be an obligate precursor for IBC, one would expect a similar prevalence of HER2-positivity in both invasive and in situ carcinoma, since HER2-positivity embodies a survival benefit for cancer cells [11]. The marked difference in HER2-positivity rates between DCIS and IBC implies that *HER2* amplification is an early oncogenic event, acting as a driver of neoplastic cell proliferation rather than as an instigator of transition from in situ to invasive carcinoma [13]. An accumulation of other (yet unknown) oncogenic events, possibly in association with tumor microenvironmental factors, might subsequently trigger the transition to invasion [14–16]. An additional argument favoring the ‘driver theory’ is the high rate (> 90%) of HER2-positivity in mammary Paget’s disease, suggesting that HER2 is essential for intraductal and intraepithelial spread of the neoplastic cells [17].

An alternative theory suggests that HER2-negative subclones containing other oncogenic drivers outgrow the HER2-positive DCIS cells, eventually resulting in HER2-negative IBC. This ‘negative selection’ phenomenon was observed in patients with clonally related HER2-positive DCIS and HER2-negative ipsilateral invasive recurrence within a genomic analysis by the Grand Challenge PRECISION Consortium [18]. The explanation of the HER2 paradox in breast cancer is limited to theories deduced from observational, mostly retrospective data. Further studies are required to fully elucidate this discrepancy.

## Is HER2-positive DCIS associated with an increased risk of subsequent IBC?

The natural history of DCIS is poorly understood, as most patients undergo surgery after its diagnosis, which prevents to study its natural course [1]. Data on the progression risk of DCIS to IBC are retrospectively derived from women who were diagnosed with DCIS but who did not undergo (immediate) surgery for variable reasons, such as the Forget-Me-Not 1 and 2 studies [19, 20]. This specific patient population, often presenting comorbidities, renders such studies prone to bias. Interestingly, the 10 year cumulative risk of ipsilateral IBC in the Forget-Me-Not 2 study was not substantially different between intermediate and high grade DCIS, although it was significantly lower for low grade DCIS [19]. The HER2 status was not available in this study, but larger DCIS size was associated with increased ipsilateral IBC risk, regardless of DCIS grade [19]. As HER2-positive DCIS are generally larger than HER2-negative DCIS (Table 1) [7, 11, 12, 21–24], it could be worthwhile to retrospectively determine the HER2 status in the Forget-Me-Not 2 study, to investigate its association with subsequent ipsilateral IBC. Interestingly, retrospective HER2 IHC within the patient cohort of the UK/ANZ DCIS randomized trial demonstrated that HER2-positive DCIS were more frequently associated with ipsilateral recurrence than HER2-negative DCIS (30.2% versus 15.2%, respectively), but these recurrences were less often IBC (28.4% versus 46.5%, respectively) [12]. In other words, the risk of ipsilateral recurrence is much higher in HER2-positive DCIS, but once ipsilateral recurrence occurs, it is less likely to be IBC [12].

**Table 1:** HER2 status generally correlates with DCIS size, with HER2-positive DCIS often being larger than HER2-negative DCIS in most studies

References	Publication year	Total number of DCIS lesions included	Size HER2- DCIS (mm)	Size HER2 + DCIS (mm)	Reported *p* value (univariate analysis)
Van Bockstal et al. [22]	2014	89	30.9 ± 34.6	29.6 ± 23.8	0.544
Borgquist et al. [7]	2015	409	22.8% of DCIS > 25 mm	38.7% of DCIS > 25 mm	0.002
Williams et al. [20]	2015	314	Lum A: 15.8 TN: 15.0	HR + : 20.0 HR-: 24.5	0.005
Miligy et al. [23]	2019	646	119 (23%) are larger than 40 mm	47 (37%) are larger than 40 mm	< 0.0001
Thorat et al. [12]	2021	713	Median size HER2 IHC 0/1 + : 13.8 mm Median size HER2 IHC 2 + : 13.5 mm	Median size: 16 mm	0.0001
O’Keefe et al. [11]	2021	1540	26% of DCIS is > 15 mm	33% of DCIS is > 15 mm	0.0142
Yang et al. [24]	2022	5.628	72% of DCIS is < 16 mm	64% of DCIS is < 16 mm	< 0.001

*DCIS* ductal carcinoma in situ, *HR* hormone receptor, *lum* luminal, *neg* negative, *pos* positive, *TN* triple negative

As for the use of HER2 status as a prognostic marker for the overall ipsilateral recurrence risk after surgery, the presently available data are contradictory [25]. Some studies identified a correlation between HER2-positive DCIS and increased ipsilateral in situ recurrence risk [7, 8], whereas others observed an association between HER2-positive DCIS and increased ipsilateral invasive recurrence risk (Table 2) [7, 8, 11, 12, 26–34]. Available literature on this topic has been recently reviewed by Garg and Thorat [35], and by Akrida and Mulita [25]. The lack of a significant association between HER2 status and ipsilateral recurrence risk is likely due to lack of power, as many retrospective studies were performed on cohorts of limited size. Many studies were therefore unable to perform reliable multivariable analysis. However, treatment-related confounding in real-world cohorts, outside the clinical trial setting, probably plays an important role as well, given the important radiotherapy benefit observed in HER2-positive DCIS [12, 35]. When adjuvant therapy is not randomly allocated, HER2-positive DCIS are much more likely to be irradiated, since these lesions more frequently present with unfavorable histopathological characteristics, such as high nuclear grade, large size, and necrosis [7, 21, 22, 35]. Without knowledge of the pretreatment HER2 status, real-world DCIS patient cohorts, either prospectively or retrospectively investigated, suffer from a substantial treatment bias [35]. Future routine HER2 assessment could lower the threshold for adjuvant radiotherapy, as a retrospective analysis of the UK/ANZ DCIS randomized trial population showed a higher benefit from adjuvant radiotherapy in HER2-positive DCIS than in HER2-negative DCIS [12]. Vice versa, de-escalation of the current DCIS treatment by omitting radiotherapy could be considered in ER-positive, HER2-negative low grade DCIS [12], resulting in more personalized treatment.

**Table 2:** Non-exhaustive overview of studies reporting on the association between HER2 status of DCIS and the ipsilateral overall, in situ and invasive recurrence risk after initial treatment for DCIS

References	Pub year	Total N° DCIS included	Length of follow-up (median or mean)	Treatment	Number of overall recurrences	Number of in situ recurrences	Number of invasive recurrences	In situ recurrence (HR and/or p-value)	Invasive recurrence (HR and/or p-value)	Overall recurrence (HR and/or *p* value)
Ringberg et al. [26]	2001	121	62 months	BCS without RT	31 (26%)	18 (15)	13 (11)	–	–	1.7 (0.8–3.6) *p* = 0.20
Provenzano et al. [27]	2003	95	101 months	BCS with or without RT and/or HT	53 cases (56%)	28 (29%)	23 (24%)	NS	NS	5 (1.4–26.9) *p* = 0.008
Barnes et al. [28]	2005	129	5 years	BCS with or without RT, or mastectomy	29 cases (30%)	28 (22%)	11 (9%)	–	–	*p* = 0.0102
Nofech-Mozes et al. [29]	2009	133	8.9 years	BCS without RT	41/133 (30.8%)	20 (15%)	21 (15.8%)	–	–	1.927 (1.16–3.653) *p* = 0.044
Kerlikowske et al. [30]	2010	329	8.2 years	BCS without RT	143 cases (43.5%)	71 cases (21.6%)	72 cases (21.9%)	2.0 (1.2–3.2)	1.1 (0.6–1.9)	–
Holmes et al. [31]	2011	141	125 months	BCS without RT	60 (42.6%)	49 (34.8%)	11 (7.8%)	-–	–	1.82 (1.03–3.22) *p* = 0.041
Han et al. [32]	2012	180	8.7 years (no RT) 6.3 years (RT)	BCS with or without RT	47 (26.1%)	25 (13.9%)	22 (12.2%)	2.26 (1.03–4.96) *p* = 0.043	1.73 (0.74–4.07) *p* = 0.21	1.98 (1.11–3.53) *p* = 0.020
Rakovitch et al. [33]	2012	213	BCS with RT: 7.7 years BCS without RT: 8.7 years	BCS with RT (72) BCS without RT (141)	50 (23.5%)	26 (12.2%)	24 (11.3%)	2.72 (1.26–5.88) *p* = 0.01	1.58 (0.69–3.62) *p* = 0.28	2.11 (1.21–3.68) *p* = 0.01
Curigliano et al. [8]	2015	1667	7.6 years	BCS with or without RT, or mastectomy	342	141	201	1.59 (1.06–2.39) *p* = 0.01	0.94 (0.66–1.35) *p* = 0.179	1.18 (0.9–1.54) *p* = 0.437
Borgquist et al. [7]	2015	420	184 months	Mastectomy, or BCS with or without RT No HT	95	49	46	1.63 (0.92–2.89) *p* = 0.09	0.78 (0.40–1.55) *p* = 0.48	1.20 (0.78–1.85) *p* = 0.40
Visser et al [34]	2018	674	12.0 years	BCS alone	200 (nested case–control study)	0	200	–	1.56 (1.05–2.31) *p* = 0.03	–
O’Keefe et al. [11]	2021	1540	44.5 months	RT	23 (1.5%)	1 (0.1%)	22 (1.4%)	–	1.96 (0.84–4.58) *p* = 0.12	–
Thorat et al. [12]	2021	713	12.7 years	BCS with or without RT and/or HT	376	197	162	2.90 (1.91–4.40) *p* = 0.0003	1.40 (0.81–2.42) *p* = 0.55	2.27 (1.64–3.14) *p* = 0.0004

*BCS* breast-conserving surgery, *DCIS* ductal carcinoma in situ, *HR* hazard ratio, *HT* hormonal therapy, *NS* not significant, *RT* radiotherapy

## Is HER2-positive DCIS always associated with HER2-positive IBC?

An alternative method to analyze the spontaneous progression to IBC, is to study only those DCIS patients who developed an ipsilateral recurrence after breast-conserving surgery [1]. Most ipsilateral recurrences in the randomized EORTC-10853 trial developed within the same quadrant as the primary DCIS [36]. A substantial percentage of these primary and recurrent lesions showed similar histo-morphological and immunohistochemical profiles, suggesting that most ipsilateral recurrences represent outgrowths of residual, initially incompletely removed DCIS [36]. In a series of 266 DCIS patients with ipsilateral recurrences, invasive recurrences were more often preceded by ER-positive, HER2-negative DCIS, whereas in situ recurrences were more often preceded by ER-negative, HER2-positive DCIS [37]. Discordant HER2 status occurred only in 10,5% of cases and was more frequently observed in invasive recurrences [38]. According to Visser et al., around one in three HER2-positive DCIS with an ipsilateral invasive recurrence shows a discordant HER2 status [39]. Similar discordant HER2 status rates were observed by Gennaro et al. [40]. Although histomorphology and immunohistochemical profiles can hint at clonality between primary DCIS and recurrent tumors, extensive molecular analysis is required to establish a strong conclusion. Gorringe et al. used copy number analysis to study the clonal relationship between eight primary DCIS and their ipsilateral recurrences, and six tumors showed clear copy number events suggesting clonality [41]. The most extensive genomic analysis so far was performed by the Grand Challenge PRECISION Consortium, comprising 34 DCIS with in situ recurrence and 95 DCIS with invasive recurrence [18]. Clonality between primary DCIS and its recurrence was formally established for approximately 75% of patients, and despite this clear clonal relationship, some recurrences showed a discordant HER2 status [18]. It would be interesting to investigate whether HER2-positive primary DCIS is more frequently observed in patients with ipsilateral clonally related invasive recurrences, regardless of the HER2 status of this recurrence, since the main purpose of surgical DCIS treatment is to prevent IBC development, and thus, risk of systemic disease and death [1].

## Can HER2-positivity be used as a predictive marker?

At present, HER2 status cannot be used as a predictive marker for response to anti-HER2 targeted therapies in DCIS, due to lack of sufficient evidence. So far, only one randomized controlled clinical trial has investigated the effect of trastuzumab in a large cohort of DCIS patients: the NSABP B-43 trial did not show a significant benefit from two doses of adjuvant trastuzumab in pure DCIS patients treated with breast-conserving surgery and adjuvant radiotherapy [42]. There was a statistically nonsignificant reduction of 19% of the ipsilateral recurrences in favor of trastuzumab, but the foreseen objective of a 36% reduction was not met [42]. This observed difference could be due to a lack of power whereas a clinical effect is present, due to insufficient follow-up, or due to chance. Longer follow-up within the NSABP B-43 trial cohort would be interesting to investigate late treatment effects.

An open-label phase 2 trial, including 24 patients with HER2-positive DCIS, investigated whether preoperative single-dose intravenous trastuzumab could evoke a therapy response [43]. Despite the absence of a histopathological response, treated patients showed higher numbers of CD56-positive natural killer cells, hinting at increased antibody-dependent cell-mediated cytotoxicity [43]. These non-significant results might be due to the limited number of doses of trastuzumab administered, as DCIS admixed with HER2-positive IBC often shows substantial regression after neoadjuvant treatment [44]. Future studies could explore whether prolonged preoperative monotherapy with trastuzumab could downsize pure DCIS, aiming to reduce both the ipsilateral recurrence risk and the resected volume during breast-conserving surgery, with potentially better cosmetic outcome. On the other hand, the use of systemic anti-HER2 treatment is questionable, since DCIS is only a non-obligate precursor of invasive breast cancer, resulting in potential overtreatment for the majority of HER2-positive DCIS patients.

HER2 status could be used as a predictive marker for response to radiotherapy [35]. A retrospective analysis, performed on available tissue samples within the prospective UK/ANZ DCIS Randomized Trial, is the only large-scale study to date which performed HER2 IHC on a patient cohort with random allocation to adjuvant radiotherapy [12]. Thorat et al. demonstrated that HER2-positive DCIS patients substantially benefited from adjuvant radiotherapy in comparison with HER2-negative DCIS patients, with a greater reduction in in situ recurrences, but not in invasive recurrences [12]. Ipsilateral in situ recurrence was reduced by 84% by adjuvant radiotherapy in the HER2-positive DCIS patient group, whereas this reduction amounted only to 42% in the HER2-negative DCIS patients [12, 35]. Radiotherapy resulted in similar ten-year ipsilateral recurrence rates in HER2-positive (11.0%) and HER2-negative (9.6%) DCIS patients, whereas omission of radiotherapy resulted in much higher ten-year ipsilateral recurrence rates in HER2-positive (42.1%) than HER2-negative (17.5%) DCIS patients, mainly due to a substantial increase in in situ* recurrences* [12]. This observation fuels the hypothesis that adjuvant radiotherapy could be omitted in small hormone receptor-positive, HER2-negative DCIS, especially when margin width is at least 2 mm [45]. Given the high number of *in* situ recurrences in HER2-positive DCIS treated with lumpectomy alone, and given its excellent response to irradiation, it seems desirable to offer radiotherapy to all HER2-positive DCIS patients treated with breast-conserving surgery, to optimize local control. Patient age at diagnosis, as well as any comorbidities, should likely be taken into account too, since adjuvant radiotherapy after breast-conserving surgery for DCIS does not affect overall survival. Nevertheless, systematic HER2 IHC in daily practice seems therefore helpful to offer personalized therapy to DCIS patients [35].

## Can HER2 status be useful for preoperative work-up?

Several studies have shown that HER2-positivity in pure DCIS is strongly associated with larger DCIS size (Table 1) [7, 12, 21, 23]. Similarly, HER2-positive IBC is more often associated with a DCIS component than HER2-negative IBC, and this DCIS component is significantly larger and more frequently associated with positive margins [46, 47]. Interestingly, Zhou et al. reported that larger (> 15 mm) primary DCIS lesions were more frequently associated with an in situ recurrence than with an invasive recurrence [37]. All these observations indirectly corroborate the underlying cause of the HER2 paradox, i.e. HER2 is a potent driver of cancer cell proliferation instead of cancer cell invasion [13]. Such powerful neoplastic proliferation can then colonize and involve a complete breast lobe, supporting the ‘sick lobe theory’ described by Tibor Tot [48]. If HER2-positive DCIS is larger, the risk of positive margins is higher, and therefore, the risk of incompletely surgically removed DCIS is higher. The residual DCIS in the breast can then continue to proliferate, slowly but steadily spreading throughout the affected ‘sick lobe’. This could explain why several retrospective studies observed a significant association between HER2-positivity and increased ipsilateral in situ recurrence risk [7, 8, 12]. The number of in situ recurrences in the NSABP B-43 trial doubled the number of invasive recurrences [42], which provides further indirect support for this theory. Preoperative assessment of the HER2 status in biopsy-diagnosed pure DCIS could encourage breast surgeons to perform wider local excisions for HER2-positive DCIS, thereby aiming to reduce the risk of involved margins and ipsilateral (in situ) recurrence risk. Once the results of four ongoing active surveillance trials will be available, watchful waiting might even become a legitimate option for ER-positive, HER2-negative non-high grade DCIS patients [49].

In addition, HER2-positivity in pure biopsy-diagnosed DCIS is associated with increased upstaging to invasive carcinoma after subsequent surgery [50–52]. Pre-operative knowledge of the HER2 status of DCIS could therefore help in the selection of patients with a potential benefit from axillary staging by sentinel node procedure.

## Could HER2 IHC improve DCIS grading and diagnostic quality?

It is a commonly acknowledged fact that grading of DCIS is subject to substantial interobserver variability [2, 53, 54]. During the past decades, DCIS grading was entirely based upon histo-morphological evaluation of cytonuclear atypia [2]. Some classification systems also included a particular architecture and/or comedonecrosis, but *grosso modo*, their main histopathological constituents are similar. As pathologists are not computers, it is challenging to objectively categorize the biological continuum of cytonuclear atypia into three categories [2]. Interestingly, the majority-based opinion regarding DCIS grade among 38 pathologists is associated with the risk of ipsilateral IBC development [54].

**Fig. 3:**
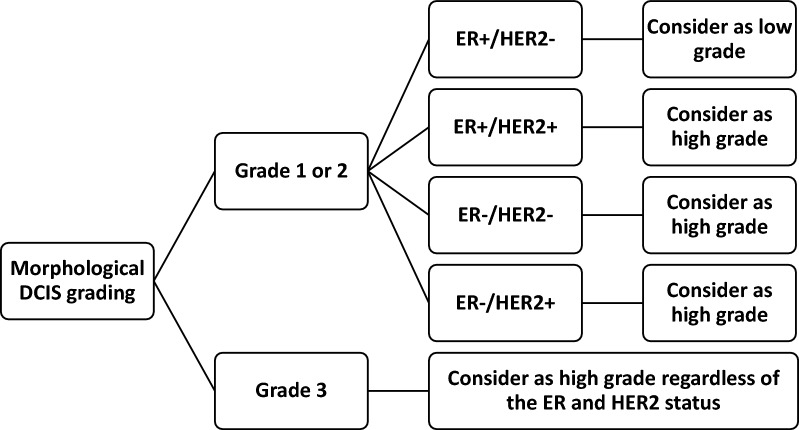
Flowchart for integrated morphological and immunohistochemical grading. The implementation of routine ER and HER2 assessment might have added value to morphological DCIS grading, which is prone to inter-observer variability. A similar workflow is implemented in the ongoing COMET active surveillance trial

Since HER2 protein overexpression in DCIS is strongly associated with high grade atypia (Table 3) [7–9, 11, 12, 21–23, 26, 31, 42, 55], Van Seijen et al. investigated the addition of HER2 IHC to the reproducibility of histopathological grading [56]. Low grade DCIS is unlikely to present with HER2-positivity. For example, the NSABP B-43 cohort of 2.014 HER2-positive DCIS contained only twenty low grade DCIS (1%) and 317 intermediate grade DCIS (16%) [42]. Although not all high grade DCIS present with HER2 protein overexpression, a 3 + HER2-positive score is very suggestive of high grade (Table 3) [7, 11, 12, 21–23, 26, 31, 42, 55, 56]. HER2 IHC is also prone to a certain degree of interobserver variability, but this appears to be mainly an issue for the distinction of 0 scores versus 1 + /2 + scores, whereas the identification of HER2 3 + cases is more reproducible [57]. The systematic use of HER2 IHC in the histopathological work-up of DCIS could therefore improve the reproducibility of grading, which is an important prognostic factor to identify those patients at risk of developing a second ipsilateral breast tumor, either in situ or invasive. This practice is already standard in currently ongoing active surveillance trials LORD and COMET [49, 58]. The addition of HER2 IHC to the histopathological work-up of DCIS calls for new guidelines. We propose an integration of morphological features and ER and HER2 IHC in Fig. 3, reflecting the workflow of the currently ongoing COMET trial [58]. The feasibility of this integration likely requires prospective validation before routine implementation.

**Table 3:** Non-exhaustive overview of the association between HER2 status and nuclear grade of DCIS

References	Publication year	Total N° DCIS included in the study	Proportion high Grade in HER2- DCIS	Proportion high grade in HER2 + DCIS	Reported *p* value
Ringberg et al. [26]	2001	187	27/86 (31%)	69/101 (68%)	*P* < 0.003
Meijnen et al. [55]	2008	163	20/99 (20%)	52/64 (81%)	*P* < 0.001
Holmes et al. [31]	2011	141	17/102 (17%)	24/39 (62%)	*P* < 0.001
Van Bockstal et al. [22]	2014	89	10/46 (22%)	27/43 (63%)	*P* < 0.001
Borgquist et al. [7]	2015	420	109/220 (49.5%)	111/220 (46%)	*P* < 0.001
Curigliano et al. [8]	2015	1667	142 (12.8%)	336 (60%)	*P* < 0.001
Williams et al. [21]	2015	314	96/175 (54.9%)	106/139 (76%)	*P* < 0.001
Miligy et al. [23]	2019	646	278/518 (54%)	115/128 (90%)	*P* < 0.0001
Cobleigh et al. [42]	2021	2014	–	1677/2014 (83.7%)	–
Thorat et al. [12]	2021	713	287/414 (69.3%)	204/221 (92.3%)	*P* < 0.001
O’Keefe et al. [11]	2021	1540	356/1123 (31.7%)	302/417 (72.4%)	*P* < 0.001

*DCIS* ductal carcinoma in situ

## Which practical issues need to be considered for routine HER2 IHC?

As shown in the SWOT analysis (Fig. 1), routine IHC for ER, PR and HER2 in DCIS will increase the working costs for pathology labs. However, these immunohistochemical profiles have the potential to lead towards personalized treatment. If some DCIS patients could forego adjuvant radiotherapy, or even surgery by opting for active surveillance, the routine implementation of IHC could perhaps reduce therapy-related costs. At present, it is difficult to provide a detailed cost/benefit analysis, as we did not yet obtain the data of ongoing active surveillance trials to decide how many patients could forego surgery [49, 58]. Once these data are available, such a health economic analysis could be undertaken. Nevertheless, this remains a difficult financial assessment, as reimbursement of health care-related costs differs between countries. Moreover, it is yet unknown how active surveillance needs to be performed: which type of investigation is required and what is its frequency? From a health economic point of view, it might even be cheaper to perform upfront surgery, instead of offering regular medical imaging with associated biopsies. This question needs to be addressed in future clinical and health economic studies.

Additionally, the question remains whether HER2 2 + DCIS need to undergo complementary analysis by in situ hybridization (ISH), as is currently performed for invasive breast cancer [59]. Performing ISH additionally increases the cost for histopathology labs. The only large-scale randomized clinical trial on DCIS wherein HER2 IHC has been performed in a systematic way in a central laboratory is the NSABP B-43 study [9]. Here, ISH was performed on all centrally stained HER2 1 + and 2 + DCIS. In total, 1424 out of 5645 DCIS were 1 + (25,2%) of which only one was amplified) [9]. It therefore seems not necessary to perform ISH testing on HER2 1 + cases. In NSABP B-43, 437 patients out of 5645 (7,7%) had a HER2 2 + score, of which 91 DCIS were amplified (20,8%). In other words, the IHC 2 + amplified DCIS represented only 1,6% of that total DCIS population [9]. It remains to be investigated whether the HER2 amplification in these HER2 2 + DCIS has an important biological and clinical consequence, but until we have large-scale studies that can reliably provide these data, we could extrapolate the ASCO/CAP algorithm for HER2 assessment in invasive breast cancer to DCIS, and perform ISH on all DCIS with a HER2 2 + score.

Lastly, the question remains whether IHC for PR is required. In several countries, patients with hormone receptor-positive DCIS are eligible for endocrine therapy, and IHC for ER and PR is often performed simultaneously. There is no strong evidence available to support this practice in DCIS; it is mainly based on extrapolation of the ASCO/CAP algorithm for hormonal receptor status assessment in invasive breast cancer patients, although the ASCO/CAP expert panel considers PR IHC as optional [60]. Patients with ER-positive, PR-positive invasive breast cancer tend to respond better to hormonal therapy than patients with an ER-positive, PR-negative invasive breast cancer [60], but there is no proof of such benefit in DCIS. A retrospective analysis of DCIS samples of patients enrolled in the NSABP B-24 trial showed no added value of PR IHC to ER IHC [61]. Patient stratification by PR status alone or by combined ER and PR status was not more predictive for response to endocrine therapy than ER status alone [61]. We propose thus to follow the ASCO/CAP expert panel consensus, which considers PR IHC as optional but not obligatory in DCIS.

## Conclusions

Hormone receptor status in pure DCIS is already routinely assessed in many countries, but the evaluation of HER2 status is generally omitted. We believe that systematic implementation of immunohistochemistry for ER, PR and HER2 could substantially improve the diagnostic work-up of pure DCIS, at the very least in clinical trials, but preferentially in routine practice too. HER2 immunohistochemistry (and if required, HER2 in situ hybridization for equivocal cases) signify an additional cost and increased workload for pathologists, but there are also several advantages (Fig. 1) [25, 35]. Firstly, the HER2 status in DCIS seems to be associated with ipsilateral recurrence risk. Secondly, HER2-positive DCIS tends to be larger, with a higher risk of involved margins after breast-conserving surgery, and a higher benefit from adjuvant radiotherapy. HER2-positivity in pure biopsy-diagnosed DCIS is associated with increased upstaging to invasive carcinoma after subsequent surgery. Thirdly, immunohistochemistry could reduce the present interobserver variability in morphological DCIS grading among pathologists, as HER2-positivity strongly correlates with high grade. Reproducible grading will become more important in the future, if active surveillance would enter routine practice as a legitimate alternative for surgery in low-risk DCIS patients. Routine assessment of ER, PR and HER2 status in pure DCIS is therefore a promising instrument that could facilitate the development of evidence-based and DCIS subtype-dependent guidelines, aiming to de-escalate therapy in low-risk patients.

## Data Availability

No datasets were generated or analysed during the current study.

## Abbreviations

DCIS: Ductal carcinoma in situ
EORTC: European Organization for Research and Treatment of Cancer.
ER: Estrogen receptor
HER2: Human epidermal growth factor receptor 2
IBC: Invasive breast cancer
IHC: Immunohistochemistry
ISH: In situ Hybridization
NSABP: National Surgical Adjuvant Breast and Bowel Project
PR: Progesterone receptor
SWOT: Strength, weakness, opportunity and threat

## References

[1] Van Bockstal MR, Agahozo MC, Koppert LB, van Deurzen CHM. A retrospective alternative for active surveillance trials for ductal carcinoma in situ of the breast. Int J Cancer. 2020;146:1189–97. 31018242 10.1002/ijc.32362 PMC7004157

[2] Van Bockstal MR, Berlière M, Duhoux FP, Galant C. Interobserver variability in ductal carcinoma in situ of the breast. Am J Clin Pathol. 2020;154:596–609. 32566938 10.1093/ajcp/aqaa077

[3] Margolese RG, Cecchini RS, Julian TB, Ganz PA, Costantino JP, Vallow LA, et al. Anastrozole versus tamoxifen in postmenopausal women with ductal carcinoma in situ undergoing lumpectomy plus radiotherapy (NSABP B-35): a randomised, double-blind, phase 3 clinical trial. The Lancet. 2016;387:849–56. 10.1016/S0140-6736(15)01168-X PMC4792688 26686957

[4] Forbes JF, Sestak I, Howell A, Bonanni B, Bundred N, Levy C, et al. Anastrozole versus tamoxifen for the prevention of locoregional and contralateral breast cancer in postmenopausal women with locally excised ductal carcinoma in situ (IBIS-II DCIS): a double-blind, randomised controlled trial. The Lancet. 2016;387:866–73. 10.1016/S0140-6736(15)01129-0 PMC4769326 26686313

[5] Staley H, McCallum I, Bruce J. Postoperative tamoxifen for ductal carcinoma in situ. Cochrane Database Syst Rev. 2012. 10.1002/14651858.CD007847.pub2. 23076938 10.1002/14651858.CD007847.pub2 PMC11955262

[6] Rüschoff J, Lebeau A, Kreipe H, Sinn P, Gerharz CD, Koch W, et al. Assessing HER2 testing quality in breast cancer: variables that influence HER2 positivity rate from a large, multicenter, observational study in Germany. Mod Pathol. 2017;30:217–26. 27767099 10.1038/modpathol.2016.164

[7] Borgquist S, Zhou W, Jirström K, Amini RM, Sollie T, Sørlie T, et al. The prognostic role of HER2 expression in ductal breast carcinoma in situ (DCIS); a population-based cohort study. BMC Cancer. 2015;15:1–10. 26062614 10.1186/s12885-015-1479-3 PMC4464713

[8] Curigliano G, Disalvatore D, Esposito A, Pruneri G, Lazzeroni M, Guerrieri-Gonzaga A, et al. Risk of subsequent in situ and invasive breast cancer in human epidermal growth factor receptor 2-positive ductal carcinoma in situ. Ann Oncol. 2015;26:682–7. 25600567 10.1093/annonc/mdv013

[9] Siziopikou KP, Anderson SJ, Cobleigh MA, Julian TB, Arthur DW, Zheng P, et al. Preliminary results of centralized HER2 testing in ductal carcinoma in situ (DCIS): NSABP B-43. Breast Cancer Res Treat. 2013;142:415–21. 24202240 10.1007/s10549-013-2755-z PMC4962781

[10] Schiza A, Thurfjell V, Stenmark Tullberg A, Olofsson H, Lindberg A, Holmberg E, et al. Tumour-infiltrating lymphocytes add prognostic information for patients with low-risk DCIS: findings from the SweDCIS randomised radiotherapy trial. Eur J Cancer. 2022;168:128–37. 35236568 10.1016/j.ejca.2022.01.016

[11] O’Keefe TJ, Blair SL, Hosseini A, Harismendy O, Wallace AM. HER2-Overexpressing ductal carcinoma in situ associated with increased risk of ipsilateral invasive recurrence, receptor discordance with recurrence. Cancer Prev Res. 2020;13:761–71. 10.1158/1940-6207.CAPR-20-0024 PMC7483601 32493703

[12] Thorat MA, Levey PM, Louise Jones J, Pinder SE, Bundred NJ, Fentiman IS, et al. Prognostic and predictive value of HER2 expression in ductal carcinoma in situ: results from the UK/ANZ DCIS randomized trial. Clin Cancer Res. 2021;27:5317–24. 34380636 10.1158/1078-0432.CCR-21-1239 PMC7612534

[13] Van Bockstal M, Libbrecht L, Floris G, Lambein K, Pinder S. Stromal inflammation, necrosis and HER2 overexpression in ductal carcinoma in situ of the breast: another causality dilemma? Ann Oncol. 2017;28:2317. 28911076 10.1093/annonc/mdx253

[14] Weeden CE, Hill W, Lim EL, Grönroos E, Swanton C. Impact of risk factors on early cancer evolution. Cell. 2023;186:1541–63. 37059064 10.1016/j.cell.2023.03.013

[15] Strand SH, Rivero-Gutiérrez B, Houlahan KE, Seoane JA, King LM, Risom T, et al. Molecular classification and biomarkers of clinical outcome in breast ductal carcinoma in situ: analysis of TBCRC 038 and RAHBT cohorts. Cancer Cell. 2022;40:1521-1536.e7. 36400020 10.1016/j.ccell.2022.10.021 PMC9772081

[16] Risom T, Glass DR, Averbukh I, Liu CC, Baranski A, Kagel A, et al. Transition to invasive breast cancer is associated with progressive changes in the structure and composition of tumor stroma. Cell. 2022;185:299-310.e18. 35063072 10.1016/j.cell.2021.12.023 PMC8792442

[17] Sek P, Zawrocki A, Biernat W, Piekarski JH. HER2 molecular subtype is a dominant subtype of mammary Paget’s cells. An immunohistochemical study. Histopathology. 2010;57:564–71. 20955381 10.1111/j.1365-2559.2010.03665.x

[18] Lips EH, Kumar T, Megalios A, Visser LL, Sheinman M, Fortunato A, et al. Genomic analysis defines clonal relationships of ductal carcinoma in situ and recurrent invasive breast cancer. Nat Genet. 2022;54:850–60. 35681052 10.1038/s41588-022-01082-3 PMC9197769

[19] Maxwell AJ, Hilton B, Clements K, Dodwell D, Dulson-Cox J, Kearins O, et al. Unresected screen-detected ductal carcinoma in situ: Outcomes of 311 women in the Forget-Me-Not 2 study. Breast. 2022;61:145–55. 34999428 10.1016/j.breast.2022.01.001 PMC8753270

[20] Maxwell AJ, Clements K, Hilton B, Dodwell DJ, Evans A, Kearins O, et al. Risk factors for the development of invasive cancer in unresected ductal carcinoma in situ. Eur J Surg Oncol. 2018;44:429–35. 29398324 10.1016/j.ejso.2017.12.007

[21] Williams KE, Barnes NLP, Cramer A, Johnson R, Cheema K, Morris J, et al. Molecular phenotypes of DCIS predict overall and invasive recurrence. Ann Oncol. 2015;26:1019–25. 25678586 10.1093/annonc/mdv062

[22] Van Bockstal M, Lambein K, Denys H, Braems G, Nuyts A, Van den Broecke R, et al. Histopathological characterization of ductal carcinoma in situ (DCIS) of the breast according to HER2 amplification status and molecular subtype. Virchows Arch. 2014;465:275–89. 24973889 10.1007/s00428-014-1609-3

[23] Miligy IM, Toss MS, Gorringe KL, Lee AHS, Ellis IO, Green AR, et al. The clinical and biological significance of HER2 over-expression in breast ductal carcinoma in situ: a large study from a single institution. Br J Cancer. 2019;120:1075–82. 31065110 10.1038/s41416-019-0436-3 PMC6738110

[24] Yang L, Shen M, Qiu Y, Tang T, Bu H. Molecular subtyping reveals uniqueness of prognosis in breast ductal carcinoma in situ patients with lumpectomy. Breast. 2022;64:1–6. 35462343 10.1016/j.breast.2022.03.019 PMC9039875

[25] Akrida I, Mulita F. The clinical significance of HER2 expression in DCIS. Med Oncol. 2023;40:1. 10.1007/s12032-022-01876-9 36352293

[26] Ringberg A, Anagnostaki L, Anderson H, Idvall I, Fernoë M. Cell biological factors in ductal carcinoma in situ (DCIS) of the breast-relationship to ipsilateral local recurrence and histopathological characteristics. Eur J Cancer. 2001;37:1514–22. 11506959 10.1016/S0959-8049(01)00165-4

[27] Provenzano E, Hopper JL, Giles GG, Marr G, Venter DJ, Armes JE. Biological markers that predict clinical recurrence in ductal carcinoma in situ of the breast. Eur J Cancer. 2003;39:622–30. 12628841 10.1016/S0959-8049(02)00666-4

[28] Barnes NLP, Khavari S, Boland GP, Cramer A, Knox WF, Bundred NJ. Absence of HER4 expression predicts recurrence of ductal carcinoma in situ of the breast. Clin Cancer Res. 2005;11:2163–8. 15788662 10.1158/1078-0432.CCR-04-1633

[29] Nofech-Mozes S, Trudeau M, Kahn HK, Dent R, Rawlinson E, Sun P, et al. Patterns of recurrence in the basal and non-basal subtypes of triple-negative breast cancers. Breast Cancer Res Treat. 2009;118:131–7. 19189211 10.1007/s10549-008-0295-8

[30] Kerlikowske K, Molinaro AM, Gauthier ML, Berman HK, Waldman F, Bennington J, et al. Biomarker expression and risk of subsequent tumors after initial ductal carcinoma in situ diagnosis. J Natl Cancer Inst. 2010;102:627–37. 20427430 10.1093/jnci/djq101 PMC2864293

[31] Holmes P, Lloyd J, Chervoneva I, Pequinot E, Cornfield DB, Schwartz GF, et al. Prognostic markers and long-term outcomes in ductal carcinoma in situ of the breast treated with excision alone. Cancer. 2011;117:3650–7. 21319154 10.1002/cncr.25942

[32] Han K, Nofech-Mozes S, Narod S, Hanna W, Vesprini D, Saskin R, et al. Expression of HER2neu in ductal carcinoma in situ is associated with local recurrence. Clin Oncol. 2012;24:183–9. 10.1016/j.clon.2011.09.008 21958729

[33] Rakovitch E, Nofech-Mozes S, Hanna W, Narod S, Thiruchelvam D, Saskin R, et al. HER2/neu and Ki-67 expression predict non-invasive recurrence following breast-conserving therapy for ductal carcinoma in situ. Br J Cancer. 2012;106:1160–5. 22361634 10.1038/bjc.2012.41 PMC3304413

[34] Visser LL, Elshof LE, Schaapveld M, Van De Vijver K, Groen EJ, Almekinders MM, et al. Clinicopathological risk factors for an invasive breast cancer recurrence after ductal carcinoma in situ-a nested case-control study. Clin Cancer Res. 2018;24:3593–601. 29685879 10.1158/1078-0432.CCR-18-0201

[35] Garg N, Thorat MA. HER2 expression should be routinely evaluated in DCIS to avoid under or overtreatment! Oncoscience. 2023;10:1–3. 36733476 10.18632/oncoscience.572 PMC9890724

[36] Bijker N, Peterse JL, Duchateau L, Robanus-Maandag EC, Bosch CAJ, Duval C, et al. Histological type and marker expression of the primary tumour compared with its local recurrence after breast-conserving therapy for ductal carcinoma in situ. Br J Cancer. 2001;84:539–44. 11207051 10.1054/bjoc.2000.1618 PMC2363778

[37] Zhou W, Johansson C, Jirström K, Ringberg A, Blomqvist C, Amini R-M, et al. A comparison of tumor biology in primary ductal carcinoma in situ recurring as invasive carcinoma versus a new in situ. Int J Breast Cancer. 2013;2013:1–8. 10.1155/2013/582134 PMC3893751 24490077

[38] Karlsson E, Sandelin K, Appelgren J, Zhou W, Jirström K, Bergh J, et al. Clonal alteration of breast cancer receptors between primary ductal carcinoma in situ (DCIS) and corresponding local events. Eur J Cancer. 2014;50:517–24. 24275214 10.1016/j.ejca.2013.10.020

[39] Visser LL, Elshof LE, Van De Vijver K, Groen EJ, Almekinders MM, Sanders J, et al. Discordant marker expression between invasive breast carcinoma and corresponding synchronous and preceding DCIS. Am J Surg Pathol. 2019;43:1574–82. 31206365 10.1097/PAS.0000000000001306

[40] Gennaro M, Meneghini E, Baili P, Bravaccini S, Curcio A, de Santis MC, et al. High consistency between characteristics of primary intraductal breast cancer and subtype of subsequent ipsilateral invasive cancer. Tumori. 2020;106:64–9. 31446852 10.1177/0300891619867845

[41] Gorringe KL, Hunter SM, Pang JM, Opeskin K, Hill P, Rowley SM, et al. Copy number analysis of ductal carcinoma in situ with and without recurrence. Mod Pathol. 2015;28:1174–84. 26321097 10.1038/modpathol.2015.75

[42] Cobleigh MA, Anderson SJ, Siziopikou KP, Arthur DW, Rabinovitch R, Julian TB, et al. Comparison of radiation with or without concurrent trastuzumab for HER2-positive ductal carcinoma in situ resected by lumpectomy: a phase III clinical trial. J Clin Oncol. 2021;39:2367–74. 33739848 10.1200/JCO.20.02824 PMC8462554

[43] Kuerer HM, Buzdar AU, Mittendorf EA, Esteva FJ, Lucci A, Vence LM, et al. Biologic and immunologic effects of preoperative trastuzumab for ductal carcinoma in situ of the breast. Cancer. 2011;117:39–47. 20740500 10.1002/cncr.25399 PMC2997136

[44] Ploumen R, Claassens E, Kooreman L, Keymeulen K, van Kats M, Gommers S, et al. Complete response of ductal carcinoma in situ to neoadjuvant systemic therapy in HER2-positive invasive breast cancer patients: a nationwide analysis. Eur J Cancer. 2022;175:S1. 10.1016/S0959-8049(22)01350-8 PMC10361905 37395816

[45] Morrow M, Van Zee KJ, Solin LJ, Houssami N, Chavez-MacGregor M, Harris JR, et al. Society of surgical oncology-American society for radiation oncology-American society of clinical oncology consensus guideline on margins for breast-conserving surgery with whole-breast irradiation in ductal carcinoma in situ. Pract Radiat Oncol. 2016;6:287–95. 27538810 10.1016/j.prro.2016.06.011 PMC5070537

[46] van Deurzen CHM. Predictors of surgical margin following breast-conserving surgery: a large population-based cohort study. Ann Surg Oncol. 2016;23:627–33. 27590331 10.1245/s10434-016-5532-5 PMC5149558

[47] Doebar SC, van den Broek EC, Koppert LB, Jager A, Baaijens MHA, Obdeijn IMAM, et al. Extent of ductal carcinoma in situ according to breast cancer subtypes: a population-based cohort study. Breast Cancer Res Treat. 2016;158:179–87. 27318854 10.1007/s10549-016-3862-4 PMC4937080

[48] Tot T. Subgross morphology, the sick lobe hypothesis, and the success of breast conservation. Int J Breast Cancer. 2011;2011:1–8. 10.4061/2011/634021 PMC3262566 22295230

[49] Kanbayashi C, Thompson AM, Hwang E-SS, Partridge AH, Rea DW, Wesseling J, et al. The international collaboration of active surveillance trials for low-risk DCIS (LORIS, LORD, COMET, LORETTA). J Clin Oncol. 2019;37:TPS603–TPS603. 10.1200/JCO.2019.37.15_suppl.TPS603

[50] Mori K, Takeda M, Kodama Y, Kiyokawa H, Yasojima H, Mizutani M, et al. Tumor thickness and histological features as predictors of invasive foci within preoperatively diagnosed ductal carcinoma in situ. Hum Pathol. 2017;64:145–55. 28434924 10.1016/j.humpath.2017.04.004

[51] Mustafa RE, DeStefano LM, Bahng J, Yoon-Flannery K, Fisher CS, Zhang PJ, et al. Evaluating the risk of upstaging HER2-positive DCIS to invasive breast cancer. Ann Surg Oncol. 2017;24:2999–3003. 28766212 10.1245/s10434-017-5941-0

[52] Oda G, Nakagawa T, Ogawa A, Kumaki Y, Hosoya T, Sugimoto H, et al. Predictors for upstaging of ductal carcinoma in situ (DCIS) to invasive carcinoma in non-mass-type DCIS. Mol Clin Oncol. 2020;13:67–72. 32454975 10.3892/mco.2020.2036 PMC7243301

[53] Dano H, Altinay S, Arnould L, Bletard N, Colpaert C, Dedeurwaerdere F, et al. Interobserver variability in upfront dichotomous histopathological assessment of ductal carcinoma in situ of the breast: the DCISion study. Mod Pathol. 2020;33:354–66. 31534203 10.1038/s41379-019-0367-9 PMC7983551

[54] Groen EJ, Hudecek J, Mulder L, van Seijen M, Almekinders MM, Alexov S, et al. Prognostic value of histopathological DCIS features in a large-scale international interrater reliability study. Breast Cancer Res Treat. 2020;183:759–70. 32734520 10.1007/s10549-020-05816-x PMC7497690

[55] Meijnen P, Peterse JL, Antonini N, Rutgers EJT, Van De Vijver MJ. Immunohistochemical categorisation of ductal carcinoma in situ of the breast. Br J Cancer. 2008;98:137–42. 18043578 10.1038/sj.bjc.6604112 PMC2359678

[56] van Seijen M, Jóźwiak K, Pinder SE, Hall A, Krishnamurthy S, Thomas JSJ, et al. Variability in grading of ductal carcinoma in situ among an international group of pathologists. J Pathol Clin Res. 2021;7:233–42. 33620141 10.1002/cjp2.201 PMC8073001

[57] Fernandez AI, Liu M, Bellizzi A, Brock J, Fadare O, Hanley K, et al. Examination of low ERBB2 protein expression in breast cancer tissue. JAMA Oncol. 2022;8:1–4. 35113160 10.1001/jamaoncol.2021.7239 PMC8814969

[58] Hwang ES, Hyslop T, Lynch T, Frank E, Pinto D, Basila D, et al. The COMET (Comparison of Operative versus Monitoring and Endocrine Therapy) trial: a phase III randomised controlled clinical trial for low-risk ductal carcinoma in situ (DCIS). BMJ Open. 2019;9: e026797. 30862637 10.1136/bmjopen-2018-026797 PMC6429899

[59] Wolff AC, Hammond MEH, Allison KH, Harvey BE, Mangu PB, Bartlett JMS, et al. Human epidermal growth factor receptor 2 testing in breast cancer: American society of clinical oncology/college of American pathologists clinical practice guideline focused update. Arch Pathol Lab Med. 2018;142:1364–82. 29846104 10.5858/arpa.2018-0902-SA

[60] Allison KH, Hammond MEH, Dowsett M, McKernin SE, Carey LA, Fitzgibbons PL, et al. Estrogen and progesterone receptor testing in breast cancer: American society of clinical oncology/college of American pathologists guideline update. Arch Pathol Lab Med. 2020;144:545–63. 31928354 10.5858/arpa.2019-0904-SA

[61] Allred DC, Anderson SJ, Paik S, Wickerham DL, Nagtegaal ID, Swain SM, et al. Adjuvant tamoxifen reduces subsequent breast cancer in women with estrogen receptor-positive ductal carcinoma in situ: a study based on NSABP protocol B-24. J Clin Oncol. 2012;30:1268–73. 22393101 10.1200/JCO.2010.34.0141 PMC3341142

